# Assessment of glycemic control in type 2 diabetes in the Eastern Sudan

**DOI:** 10.1186/s13104-018-3480-9

**Published:** 2018-06-08

**Authors:** Saeed M. Omar, Imad R. Musa, Osman E. Osman, Ishag Adam

**Affiliations:** 1 Faculty of Medicine, Gadarif University, Gadarif, Sudan; 2 King Abdu Aziz Armed Forces Hospital at Air Base, Dhahran, Kingdom of Saudi Arabia; 3 Faculty of Medicine, Alneelain University, Khartoum, Sudan; 4 Faculty of Medicine, University of Khartoum, P. O. Box 102, Khartoum, Sudan

**Keywords:** Diabetes, Controlled, HbA1c, Sudan

## Abstract

**Objectives:**

A cross-sectional study was conducted in Gadarif, eastern Sudan to assess glycaemic control among adult patients with type 2 diabetes in eastern Sudan. Poor glycaemic control was defined as HbA1c level of ≥ 7.0%. Questionnaire was used to gathered sociodemographic and clinical characteristics.

**Results:**

A total of 339 patients (69.9% were women) were enrolled in the study. The mean age of the participants was 54.8 (12.8) years. Approximately more than two-thirds (n = 243, 71.7%) of the participants were using oral glucose control agents. A round one-fifth (22.1%) of the participants were using insulin and only 6.2% of them were using both insulin and oral glucose control agents. The rate of poor glycemic control was 71.9%. In logistic regression analyses, duration of diabetes, medications used, and the triglycerides were not associated with poor glycemic control. However, being unmarried (OR = 3.64, 95% CI 1.21–10.90), adding sugar to the drinks (OR = 1.84, 95% CI 1.11–3.05, P = 0.017) and high cholesterol level (OR = 1.01, 95% CI 1.01–1.02.) were associated with poor glycemic control. In summary the rate of uncontrolled type 2 diabetes mellitus was considerably high especially among being unmarried patients and patients who were adding sugar to the drinks.

## Introduction

Diabetes mellitus is the main endocrinopathy and is the chronic metabolic disorder that is associated with serious medical complications. The global prevalence of diabetes is rising among adults. It has been estimated that in 2017 there are 451 million people with diabetes worldwide and the number of adult patients with diabetes mellitus is expected to increase both the developing and developed countries by 69 and 20% respectively [1, 2]. Recent report showed that the prevalence of diabetes mellitus in the Africa Region range 9.7–15.4% [3]. Type 2 diabetes mellitus, comprises almost 90% burden of the disease and the remaining 10% are type 1 diabetes or gestational diabetes [3]. Recent reports have shown that diabetes and its related complications were major health problem in Sudan [4–6].

It has been shown that poor glycemic control was associated with diabetes complications, and these complications could be avoided by good diabetic control [7, 8]. Different rate and various factors (age, gender, obesity, education, exercise) have been reported to be associated with the poor glycemic control in different settings [9–13].

While there are many published data on glycemic control and its associated factors in the different African countries [14–20], there are few published data on glycemic control in Sudan and non-exist in eastern part of Sudan [21, 22]. Furthermore, recent reports have shown that diabetes and its associated complications are major health problem in Sudan [5, 6]. It is of paramount to investigate the glycemic control in Eastern Sudan so as to generate data that is necessary for both the treating physicians as well as for health care planners. The current study was conducted to assess the prevalence and associated factors for poor glycemic control diabetes in Gadarif, eastern Sudan.

## Main text

### Methods

A cross-sectional study was conducted in the university clinics at Gadarif, eastern Sudan during the period of February through August 2017. After signing an informed consent all adult (age ≥ 18 years) patients with type 2 diabetes (men and women) were enrolled. All participants were Sudanese and have the duration of the disease for 1 year or more. Participants with age less than 18 years, type 1 diabetes, recent diagnosis of diabetes (< 1 year), on dietary control only, pregnant women, patients with haemoglobinopathy, acutely ill, debilitated patients or any chronic disease that may alter HbA1c e.g. end stage renal disease were excluded.

Questionnaire was used to gathered sociodemographic characteristics [age, sex (menor women), education (≤ secondary level or > secondary levels, employment (employed or non-employed), health insurance, marital status (married or non- married), smoking (smokers were subject who smoked more than 100 cigarettes in their lives and reported any past-year smoking), alcohol consumption (one or more drink in the past month), duration of diabetes, and comorbidities (hypertension, thyroid, hyperlipidemia, and ischemic disease).

The patients’ weight and height were measured using standard procedures and body mass index (BMI) was computed as weight/height (m^2^). Fasting cholesterol and triglyceride levels were measured using enzymatic methods.

Glycaemic control status was defined according to the HbA1c target of < 7% as recommended by the American Diabetes Association for non-pregnant adults [23]. Accordingly, HbA1c level of ≥ 7.0% was defined as ‘poor glycaemic control’.

The sample size (339) was based on the rate of uncontrolled diabetes which was reported in the previous studies [21, 22] where 68.0% of patients were expected to have uncontrolled diabetes to detect a difference of 5% at α = 0.05 with a power of 80%. We assumed that 10% of the participants might not respond or have incomplete data.

#### Statistics

Data were entered into a computer using SPSS for Windows (version 20.0). The Chi square test was used to compare proportions between patients with controlled and poor glycemic control. The Kolmogorov–Smirnov test was used for testing the normality of continuous data (age, duration of diabetes, BMI, cholesterol and triglycerides levels). The continuous parametric and non-parametric data were compared by t test and Mann–Whitney test, respectively, between the two groups (controlled and poor glycemic control). Logistic regression analyses were performed with poor glycemic control as the dependent variable. Independent variables (age, sex, marital status, education, presence of comorbidity, alcohol intake, measuring blood glucose at home, having medical insurance, BMI, cholesterol and triglycerides levels) were entered into the model if their univariate P was < 0.20. Odds ratios (ORs) and 95% confidence intervals (CIs) were calculated and a P value of < 0.05 was considered significant.

### Results

A total of 339 adult patients were enrolled in the study. Among them, 237 (69.9%) patients were women. The mean (SD) age of the participants was 54.8 (12.8%) years (range 195–90 years). The majority (90.0%) of the participants were married. More than half (62.5%) of the participants were adding sugar to the drink. Few (3.7%) participants were cigarette smokers and only 3 (0.9%) patients consumed alcohol. A total of 300 (88.5%) participants had medical insurance (Table 1). The mean (SD) duration of diabetes was 6.8 (5.5%) years.

**Table 1:** Characteristics of the patients with type 2 diabetes in eastern Sudan (n = 339)

Variable	Value	Percentage
Age (years)^a^	54.8	12.8
Male sex	102	30.1
Education ≤ secondary level	259	76.4
Married	305	90.0
Employed	150	44.2
Duration of diabetes (years)^b^	5.8	3.0–10.0
Presence of comorbidity	126	37.2
Smoking/ex-smoking	13	3.9
Alcohol intake	3	0.9
Add sugar to the food	300	88.5
Oral glucose control agents	243	71.7
Insulin	75	22.1
Oral hypoglycemic and insulin	21	6.2
Measuring blood sugar at home	31	9.1
Body mass index (kg/m^2^)^b^	26.4	23.6–29.6
Fasting blood glucose (mg/dl)	149.0	120.0–192.0
Hemoglobin A1c (%)	8.6	6.9–9.9
Cholesterol (mg/dl)^b^	165.8	134.0–190.0
Triglycerides (mg/dl)^b^	127.0	90.0–160.0

^a^Mean (SD)

^b^Median (interquartile range)

Approximately more than two-thirds (n = 243, 71.7%) of the participants were using oral glucose control agents. Around one-fifth (22.1%) of the participants were using insulin and only 6.2% of them were using both insulin and oral glucose control agents. Around one-third (37.2%) of the participants had co-morbidity. The most common comorbidities were hypertension (n = 114, 33.6%), thyroid disease (n = 5, 1.5%), previous ischemic disease (n = 2, 0.6%) and renal disease (n = 5, 1.5%).

The rate of poor glycemic control was 71.9%. There were no significant differences in age, sex, education, employment, presence of comorbidity, smoking, alcohol intake, type of the treatments, measuring blood glucose at home and BMI between participants with glycemic control and participants with poor glycemic control. A significantly higher number of participants with uncontrolled diabetes were married, had longer duration of diabetes, adding sugar to the table and had higher fasting blood glucose, cholesterol and triglyceride levels compared with those with controlled diabetes (Table 2).

**Table 2:** Comparison of clinical and biochemical characteristics between patients with controlled and poor glycaemic control

Variable	Controlled diabetes (n = 96)	Uncontrolled diabetes (n = 243)	OR	95% CI	P
Age (years)^a^	54.9 (12.7)	54.7 (12.9)	0.99	0.98–1.01	0.877
Male sex	28 (29.2)	74 (30.5)	0.94	0.56–1.57	0.896
Education ≤ secondary level	19 (19.8)	61 (25.1)	1.35	0.76–2.42	0.324
Married	92 (95.8)	213 (87.7)	0.79	0.68–0.91	0.026
Employed	45 (46.9)	105 (43.2)	0.86	0.53–1.38	0.547
Duration of diabetes (years)^b^	4.0 (2.23–7.0)	6.0 (3.0–10.0)	1.05	1.01–1.11	0.014
Presence of comorbidity	41 (42.7)	85 (35.0)	0.72	0.44–1.16	0.212
Smoking/ex-smoking	4 (4.2)	9 (3.7)	2.48	0.73–8.40	0.827
Alcohol intake	119 (57.2)	101 (59.1)	1.07	0.71–1.62	0.550
Add sugar to the food	51 (53.1)	161 (66.3)	1.73	1.07–2.80	0.034
Oral hypoglycemic drugs	71 (74.0)	172 (70.8)	0.40	0.11–1.41	0.156
Insulin	22 (22.9)	53 (21.8)	0.40	0.10–1.50	0.175
Oral hypoglycemic and insulin	3 (3.1)	18 (7.4)	Ref	–	–
Measuring blood sugar at home	11 (11.5)	(8.2)	0.69	0.31–1.50	0.403
Body mass index (kg/m^2^)^b^	26.7 (23.2–29.7)	26.2 (23.6–29.4)	1.01	0.97–1.06	0.926
Fasting blood glucose	121.0 (103.03–141.7)	165.0 (130.0–216.0)	1.02	1.02 –1.04	< 0.001
Cholesterol (mg/dl)^b^	159.5 (122.0–178.2)	165.0 (138.0–194.0)	1.01	1.01–1.03	0.009
Triglycerides (mg/dl)^b^	120.0 (83.2–138.7)	130.6 (91.0–163.0	1.01	1.01–1.04	0.032

^a^Values are means (SD)

^b^Median (interquartile range)

In logistic regression analyses, duration of diabetes, drugs used, and the triglycerides were not associated with poor glycemic control. However, being unmarried (OR = 3.64, 95% CI 1.21–10.90, P = 0.021), adding sugar to the drinks (OR = 1.84, 95% CI 1.11–3.05, P = 0.017) and high cholesterol level (OR = 1.01, 95% CI 1.01–1.02, P = 0.036) were associated with poor glycemic control (Table 3).

**Table 3:** Binary regression analyses of factors related to poor glycemic control in eastern Sudan

Variable	OR	95% CI	P
Un-married	3.64	1.21–10.90	0.021
Duration of diabetes (years)	1.04	0.99–1.10	0.085
Add sugar to the food	1.84	1.11–3.05	0.017
Drug used	1.05	0.66–1.65	0.830
Cholesterol (mg/dl)^a^	1.01	1.01–1.02	0.036
Triglycerides (mg/dl)	1.01	0.99–1.01	0.170

^a^Controlled for triglyceride

## Discussion

The main findings of the current study were the high rate (71.9%) of poor glycemic control, especially among unmarried and patients who were adding sugars to the drink. This is lower than the rate (85.0%) of poor glycemic control previously reported among 387 Sudanese patients with type 2 diabetes (50.4% males and 49.6% females) [21].

Different rates of poor glycemic control were reported in the various African settings e.g. 74.0% in Cameroon and Guinea [15], 61.3% in Zambia [17], 69.7% in Tanzania [18], 75.2% in Senegal [16], 79.2% in Uganda [19], and 62% in Nigeria [20].

It has been observed that only 33.8% of patients in eastern Saudi were achieving their glycemic control target (fasting or random capillary blood glucose < 130 or < 180 mg/dL respectively). Higher age, current smoking and lower level of physical activity were the predictors for uncontrolled diabetes [24]. The current study and the later ones should be compared cautiously because some of them used the fasting glucose to assess the glycemic control while we used HbA1c to assess the glycemic control. HbA1c is a reliable standard indicator to predict the control of diabetes mellitus as it reflects status of blood sugar during last 4 weeks to months and is not affected by many factors such as acute stress or fasting state.

The current study showed that age, duration of diabetes and BMI were not associated with poor glycemic control. The lack of association between these factors and glycemic control in our study is in contrast with the findings by Kamuhabwa and Charles in Tanzania [18]. Kamuhabwa and Charles have shown the longer duration of the diabetes was associated with poor glycemic control [18]. The plausible explanation of the association between the longer duration of diabetes and the poor glycemic control is the exhaustion of the pancreas to produce more insulin. The difference in the results between our findings and the Tanzanian ones could be explain by the difference in the socio-demographic and ethnic characteristics.

Education and employment were not associated with poor glycemic control in the current study. This goes with the previous report from Sudan [22] and Tanzania [18] where education was not associated with glycemic control. It has been shown that education was positively associated with good glycemic control [14]. Education (diabetes education) could be an important tool to raise patient awareness and have a positive impact on glycaemic control.

In the current study being unmarried participants were at 3.64 higher risks to have poor glycemic control. This is in contrast with the findings of the previous study [18]. Perhaps unmarried patients might lack the adequate/sufficient care of the family or for the same reason that they were un-married and have poor glycemic control.

The current study showed that patients who were adding sugar to the drinks were 1.84 times at higher risk to have poor glycemic control. This is in line with the findings of the previous study in central Sudan [25]. The habit of adding sugar to drinks in this region of Sudan needs to be addressed further to achieve a good glycemic control. However, the dietary habits and their effects on diabetes and its control are beyond the scope of the current study.

The finding of the association between high cholesterol level and poor glycemic control (OR = 1.01, 95% CI 1.01–1.02) in our study was previously reported in Central Sudan where high plasma triglyceride, low high density lipoproteins were associated with poor glycemic control [21]. Perhaps the high level of the cholesterol among patients with poor glycemic control was the result of the poor glycemic control rather than cause. It is difficult to dissect the cause/effect relation between dyslipidaemia and poor glycemic control by cross sectional study. A longitudinal study is needed.

### Conclusion

The rate of uncontrolled type 2 diabetes mellitus was considerably high especially among unmarried patients and patients who were adding sugar to the drinks and had high cholesterol levels.

## Limitations of the study

Other factors (hemoglobinopathies, change in erythrocyte life span, ethnicity) that may have an influence on HbA1c were not investigated. Furthermore, physical activity and psychological status may have effects on glycemic control and the outcomes of diabetes care were not investigated too.

## Abbreviations

BMI: body mass index
SD: standard devotion
ORs: odds ratios
CIs: confidence intervals

## References

[1] Cho NH, Shaw JE, Karuranga S, Huang Y, da Rocha Fernandes JD, Ohlrogge AW (2018). IDF Diabetes Atlas: global estimates of diabetes prevalence for 2017 and projections for 2045. Diabetes Res Clin Pract.

[2] Al-Quwaidhi AJ, Pearce MS, Sobngwi E, Critchley JA, O’Flaherty M (2014). Comparison of type 2 diabetes prevalence estimates in Saudi Arabia from a validated Markov model against the international diabetes federation and other modelling studies. Diabetes Res Clin Pract.

[3] Global report on diabetes WHO library cataloguing-in-publication data. ISBN. 978:92–4. http://apps.who.int/iris/bitstream/handle/10665/204871/9789241565257_eng.pdf;jsessionid=BD5BD802F099F3E72591E6182C960BA5?sequence=1. Accessed 27 May 2018.

[4] Ahmed MH, Awadalla H, Elmadhoun WM, Osman M, Noor SK, Almobarak AO (2017). Prevalence and risk factors for acute coronary syndrome among sudanese individuals with diabetes: a population-based study. Cardiol Res.

[5] Awadalla H, Noor SK, Elmadhoun WM, Almobarak AO, Elmak NE, Abdelaziz SI (2017). Diabetes complications in Sudanese individuals with type 2 diabetes: overlooked problems in sub-Saharan Africa?. Diabetes Metab Syndr.

[6] Eltom MA, Babiker Mohamed AH, Elrayah-Eliadarous H, Yassin K, Noor SK, Elmadhoun WM (2017). Increasing prevalence of type 2 diabetes mellitus and impact of ethnicity in north Sudan. Diabetes Res Clin Pract.

[7] Alkout AM, Blackwell CC, Weir DM (2000). Increased inflammatory responses of persons of blood group O to *Helicobacter pylori*. J Infect Dis.

[8] Liu ZM, Ho SC (2011). The association of serum C-reactive protein, uric acid and magnesium with insulin resistance in Chinese postmenopausal women with prediabetes or early untreated diabetes. Maturitas.

[9] Yousefzadeh G, Shokoohi M, Najafipour H (2015). Inadequate control of diabetes and metabolic indices among diabetic patients: a population based study from the Kerman coronary artery disease risk study (KERCADRS). Int J Health Policy Manag.

[10] Javaid F, Iqbal B, Mahmud S, Nanan DJ, Jabbar A, Nkouibert P (2015). Uncontrolled diabetes mellitus: prevalence and risk factors among people with type 2 diabetes mellitus in an Urban District of Karachi, Pakistan. Diabetes Res Clin Pract.

[11] Ashur ST, Shah SA, Bosseri S, Fah TS, Shamsuddin K (2016). Glycaemic control status among type 2 diabetic patients and the role of their diabetes coping behaviours: a clinic-based study in Tripoli, Libya. Libyan J Med.

[12] Cheneke W, Suleman S, Yemane T, Abebe G (2016). Assessment of glycemic control using glycated hemoglobin among diabetic patients in Jimma university specialized hospital, Ethiopia. BMC Res Notes.

[13] Angamo MT, Melese BH, Ayen WY (2013). Determinants of glycemic control among insulin treated diabetic patients in Southwest Ethiopia: hospital based cross sectional study. PLoS ONE.

[14] Van de Sande M, Dippenaar H, Rutten GEHM (2007). The relationship between patient education and glycaemic control in a South African township. Prim Care Diabetes.

[15] Camara A, Baldé NM, Sobngwi-Tambekou J, Kengne AP, Diallo MM, Tchatchoua APK (2015). Poor glycemic control in type 2 diabetes in the South of the Sahara: the issue of limited access to an HbA1c test. Diabetes Res Clin Pract.

[16] BeLue R, Ndiaye K, NDao F, Ba FNN, Diaw M (2015). Glycemic control in a clinic-based sample of diabetics in M’Bour Senegal. Health Educ Behav.

[17] Musenge EM, Michelo C, Mudenda B, Manankov A (2016). Glycaemic control and associated self-management behaviours in diabetic outpatients: a hospital based observation study in Lusaka, Zambia. J Diabetes Res.

[18] Kamuhabwa A, Charles E (2014). Predictors of poor glycemic control in type 2 diabetic patients attending public hospitals in Dar es Salaam. Drug Healthc Patient Saf.

[19] Kibirige D, Atuhe D, Sebunya R, Mwebaze R (2014). Suboptimal glycaemic and blood pressure control and screening for diabetic complications in adult ambulatory diabetic patients in Uganda: a retrospective study from a developing country. J Diabetes Metab Disord.

[20] Ngwogu K, Mba IE, Ngwogu AC (2012). Glycaemic control amongst diabetic mellitus patients in Umuahia Metroppolis, Abia State, Nigeria. Int J Basic Appl Innov Res.

[21] Noor SK, Elmadhoun WM, Bushara SO, Almobarak AO, Salim RS, Forawi SA (2017). Glycaemic control in Sudanese individuals with type 2 diabetes: population based study. Diabetes Metab Syndr Clin Res Rev.

[22] Abdelgadir M, Elbagir M, Eltom M, Berne C (2006). The influence of glucose self-monitoring on glycaemic control in patients with diabetes mellitus in Sudan. Diabetes Res Clin Pract.

[23] American Diabetes Association (2013). Standards of medical care in diabetes–2013. Diabetes Care.

[24] Al-Baghli NA, Al-Turki KA, Al-Ghamdi AJ, El-Zubaier AG, Al-Ameer MM, Al-Baghli FA (2010). Control of diabetes mellitus in the Eastern Province of Saudi Arabia: results of screening campaign. East Mediterr Health J.

[25] El-Sayed EF, Awadalla H, Noor SK, Elmadhoun WM, Sulaiman AA, Almobarak AO (2017). Sugar intake in Sudanese individuals was associated with some features of the metabolic syndrome: population based study. Diabetes Metab Syndr.

